# Vaccination uptake and income inequalities within a mass vaccination campaign

**DOI:** 10.1186/s13584-019-0324-6

**Published:** 2019-07-15

**Authors:** Aviad Tur-Sinai, Rachel Gur-Arie, Nadav Davidovitch, Eran Kopel, Yael Glazer, Emilia Anis, Itamar Grotto

**Affiliations:** 1 Department of Health Systems Management, The Max Stern Yezreel Valley College, Emek Yezreel, Israel; 2 Department of Health Systems Management, School of Public Health, Faculty of Health Sciences, Ben-Gurion University of the Negev, Beer-Sheva, Israel; 3 School of Public Health, Sackler Faculty of Medicine, Tel Aviv University, Tel Aviv-Yafo, Israel; 4 Department of Medical Science, Faculty of Health Sciences, Ben-Gurion University of the Negev, Beer-Sheva, Israel; 5 Israel Ministry of Health, Jerusalem, Israel

**Keywords:** Socioeconomic status (SES), Gini inequality index, Solidarity, Mother-and-child clinic, OPV vaccination, IPV vaccination

## Abstract

**Background:**

In July 2013, Israel was swept with fear of a polio outbreak. In response to the importation of wild polio virus, the Ministry decided to take preventive action by administering oral poliovirus *vaccine* (OPV) to all children born after 1 January 2004 who had received at least one dose of inactivated poliovirus vaccine (IPV) in the past. This study analyzes the vaccination uptake rates resulting from the mass polio vaccination campaign on the basis of health inequality parameters of socioeconomic status (SES), principles of solidarity, and the Gini inequality index. The research explores understanding the value of the Gini inequality index within the context of SES and solidarity.

**Methods:**

The study is based on data gathered from the Israeli Ministry of Health’s administrative records from mother-and-child clinics across Israel. The research population is comprised of resident infants and children whom the Ministry of Health defined as eligible for the OPV between August and December 2013 (the “campaign period”). The analysis was carried out at the municipality level as well as the statistical area level.

**Results:**

The higher the SES level of the municipality where the mother-and-child clinic is located, the lower the OPV vaccination uptake is. The greater the income inequality is in the municipality where the mother-and-child clinic is situated, the lower the vaccination uptake.

**Conclusions:**

Public health professionals promoting vaccine programs need to make specially-designed efforts both in localities with high average income and in localities with a high level of income diversity/inequality. Such practice will better utilize funds, resources, and manpower dedicated to increasing vaccination uptake across varying populations and communities.

## Background

### Polio in Israel

Poliomyelitis (polio) is a highly infectious disease that can lead to paralysis and even death, caused by the polio virus infecting a person’s nervous system [1]. Polio usually affects unvaccinated children under the age of five, but can also affect adolescents and adults. There are three types of polio virus—Type 1, Type 2, and Type 3. The virus is often spread through feco-oral transmission such as drinking water contaminated with fecal matter infected with polio. No specific treatment for polio exists [1]. There are two vaccines against polio: the oral poliovirus vaccine (OPV) and the inactivated poliovirus vaccine (IPV). OPV is orally administered as drops, not requiring professional administration. IPV is an injection that requires professional administration [1]. The WHO recommends four doses of OPV between birth and 14 weeks of age, and at least one IPV dose, depending on hygiene [1, 2].

In the 1950s, Israel experienced a wave of polio that carried a 10–12% mortality rate and a 30% rate of permanent paralysis among those infected [3]. Rehabilitation services were scarce at best and generally nonexistent. By 1956, 1750 people in Israel had polio, and 85–90% of those ill were under the age of five (ibid.). After 1956, due to successful polio vaccine administration, the polio epidemic receded significantly and made way for Israel’s rehabilitation services. In 2002, along with the WHO European Region, Israel was declared a polio-free country [4]. By the end of 2004, the Israeli Ministry of Health decided to only administer the IPV polio vaccine to infants and children [4, 5]. Israel was again declared polio-free in 2010 [6]. Given the country’s collective memory of the 1950s epidemic, Israel was concerned with the potential of another polio outbreak, following several isolation of wild polio virus 1 (WPV1) in sewage in 2013.

### The 2013 polio outbreak in Israel

WPV1 was first isolated between 7 and 13 April 2013 from routine sewage treatment collection in two cities in southern Israel: Beer Sheva and Rahat [4]. As of September 1, 2013, WPV1 was detected in 87 of 220 samples from 79 sewage sampling sites in Israel collected across northern and southern Israel after February 3, 2013 [4]. The sampling coverage was then expanded to sewage sites serving as much as 80% of Israel’s population and sampling frequency was increased from monthly to weekly [5]. Most treatment-facility samples from the Southern District that were continuously WPV1 positive were from areas inhabited by Bedouin communities [4]. The presence of WPV1 in sewage-sampling sites in central Israel indicated countrywide transmission and was detected mostly around Arab or mixed Jewish-Arab communities [4]. There were only three positive sewage samples in the West Bank and one in the Gaza Strip [6].

Following the fast detection of WPV1 in Israel, the Ministry of Health adopted the addition of bivalent OPV to the basic routine IPV vaccination program [6]. The Israeli response to WPV1 was coordinated with the help of local epidemiology, infectious disease, and pediatric experts, in addition to the WHO and the US CDC [4]. Surveillance data from August 14, 2014, confirmed consistent negative results for all tested sites in Israel [5]. On April 28, 2015, the WHO recertified Israel as a polio-free country [7].

#### Health disparities/inequalities and vaccination

Public health experts have recognized socioeconomic status (SES) as “the single most important predictor variable of preventative health behavior” for more than thirty-five years [8]. Public health policy tries to implement the best possible intervention for a given target community based on modern medicine, budgetary restrictions, and politics. Such target communities are often defined by SES and/or social groups. While the terms are often used interchangeably, SES and social groups have distinct definitions. Social groups are groups that differ in their biological, social, economic, or geographical characteristics [9].

Health disparities/inequalities do not refer to all differences in health, but rather a particular type of difference in health, that usually is shaped by policy [10]. It is a difference that generally affects disadvantaged social groups disproportionately, who generally experience worse health or greater health risks than more advantaged social groups [10]. Health disparities/inequalities also address differences between varying statuses within a given population, not only “best-off” and “worst-off” populations [10]. A main indicator of health disparities/inequalities is SES. No intervention targeted at vulnerable populations can singularly address all public health goals when focusing on closing health disparities/inequalities [11]. A paradox often arises when implementing population-level interventions targeted at vulnerable populations, rooted in public health practitioners incorrectly identifying fundamental causes of diseases as well as missing social and cultural assumptions among vulnerable populations [11]. For this reason, public health interventions must be specifically tailored to vulnerable population, often in ways that would not work for non-vulnerable population.

Patterns of healthcare access and utilization vary across different SES populations [12, 13]. Still, multiple studies have tied low vaccination uptake among low SES persons and groups [14, 15]. Populations of low SES oftentimes have more economic and other social barriers to overcome in order to receive healthcare services [16–18]. Nevertheless, selected results of other studies show that this pattern is not applicable in all communities and for all types of health care services.

In particular, low-SES residents often exhibit vaccine uptake rates equal to or higher than those of higher-SES residents [19]. This pattern was evident during the 2013 Israeli polio vaccination campaign, with Binyaminy et al.’s study showing that polio vaccination uptake was higher in the minority Arab population (92%) than among the Jewish population (59%) in Israel. In addition, Binyaminy found an inverse correlation between or overall SES and polio vaccination uptake, at the municipal level among the Jewish population [20].

Our study seeks to expand on Binyaminy et al. by assessing whether vaccination uptake is related not only to the average SES level of a locality, but also to the variation in SES in a locality, as reflected in the Gini Income Index.

The Gini coefficient measures the inequality among values of frequently distribution, mainly income [21]. The closer the Gini is to 0, the smaller the health inequalities are (zero equivalent to perfect equality); as the Gini gets closer to 1, inequalities are greater (one equivalent to perfect inequality) [22]. The Gini index reveals unexpected contributors to health inequalities in different societies. In a study that used the Gini to study health inequalities in the context of vaccination in India, per-capita state domestic product and percentage of illiterate population explained 24% of total health inequalities in immunization coverage [23].

In Israel, IPV and OPV are offered at countrywide mother-and-child clinics to all clients of the Israeli healthcare system. Rates of OPV immunization had geographical variation across mother-and-child clinics in Israel.^1^ Our study recognizes the varying OPV uptake rates across Israel geographically, but focuses on uptake rates among different SES clusters, not geographical locations. Our paper analyzes the Gini inequality index in terms of (1) overall polio vaccination uptake and (2) SES status combined with vaccination uptake, to provide insights into how vaccination campaigns should be organized in order to maximize vaccination uptake, taking into account the unique circumstances of particular localities.

## Methods

Data was gathered from the administrative records of Israel’s Ministry of Health. The research population is comprised of resident infants and children whom the Ministry defined as eligible for OPV between August and December 2013 (the research population). Information about the research population was obtained on the basis of existing Ministry of Health records, which in turn were predicated on data from government mother-and-child clinics only.

The information obtained in this manner yielded focused data on several aspects during the campaign period in regard to each mother-and-child clinic around the country: the number of vaccination candidates, the number of OPV vaccinates (persons who actually received the vaccine), and segmentation of the latter population by gender and nationality: the number of boys and girls and the number of Jews, non-Jews, and persons of unknown nationality. Also available was demographic information that produced a profile of the mother-and-child clinics based on district, subdistrict, municipality, name, and address. In addition, information was provided on the number of persons who received the vaccine each day at each mother-and-child clinic around the country during the campaign period (total, segmented by gender and nationality).

After the investigation file was received, data on mother-and-child clinics (district, subdistrict, and municipality of residence; and name and address of clinic) were added in accordance with the profiling variables listed above.

Additional variables were then inserted: SES, which indicates the socio-demographic, social, and economic characteristics of the population that the mother-and-child clinics served, as well as of the municipality average SES level where the mother-and-child clinics served. The SES level of the population of a geographical unit reflects a combination of basic characteristics of the specific geographical unit investigated (for example, the population of a local authority). The concept is understood in regard to its extreme manifestations: poverty at one end of the spectrum and wealth at the other. Financial resources are a central attribute of SES, but additional elements are also correlated. This variable, calculated by the Israel Central Bureau of Statistics for statistical areas and municipalities countrywide, is based on a wide ambit of traits and criteria. The main aspects of the SES level of the residents of a geographical unit are the residents’ financial resources (from work, benefits, etc.); housing (density, quality, and other characteristics); ownership of home appliances (air conditioner, dishwasher, personal computer, etc.); motorization level (quantitative and qualitative); education; employment and unemployment characteristics; various types of socioeconomic distress; and demographic characteristics.

The last variable added was the income-inequality index, using the Gini inequality index. This variable, calculated by the Israel Central Bureau of Statistics for municipalities countrywide, is defined on a municipality level.

Once the database was completed, analysis began. The first goal of the study—profiling Israel’s 2013 polio vaccination program—was complemented by a wide and rich variety of descriptive statistical indicators. The second goal—determining the correlation between vaccination uptake among varied social groups as measured on the basis of social and economic indicators— was attained by the use of two economic indicators: the SES index (for statistical areas and municipalities) and the Gini inequality index (for municipalities).

## Results

### Aggregate analysis

**Fig. 1:**
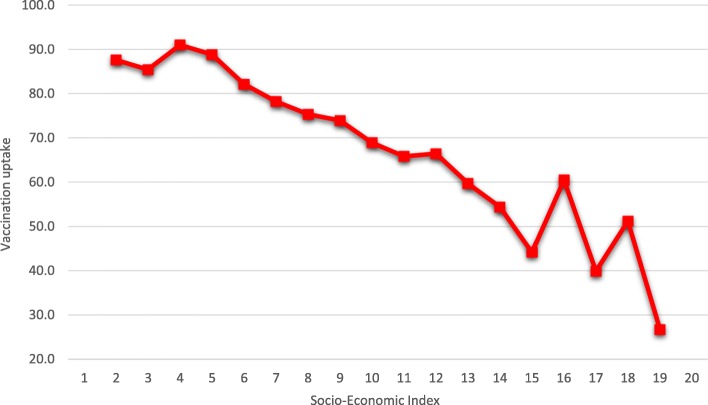
OPV Vaccination Uptake, by Socio-Economic Cluster, August–December 2013. (Statistical area, Scale 1–20). Source: Ministry of Health, processed by the authors

Figure 1 presents the correlation between the vaccination uptake rate and the SES index of the statistical area where the mother-and-child clinic is located. These variables were found to be negatively correlated, meaning that the higher the SES level of the statistical area where the mother-and-child clinic is located, the lower the vaccination uptake is. In other words, insofar as the clinic is located in a socioeconomically “better” area, the vaccination uptake in that area is lower.

**Fig. 2:**
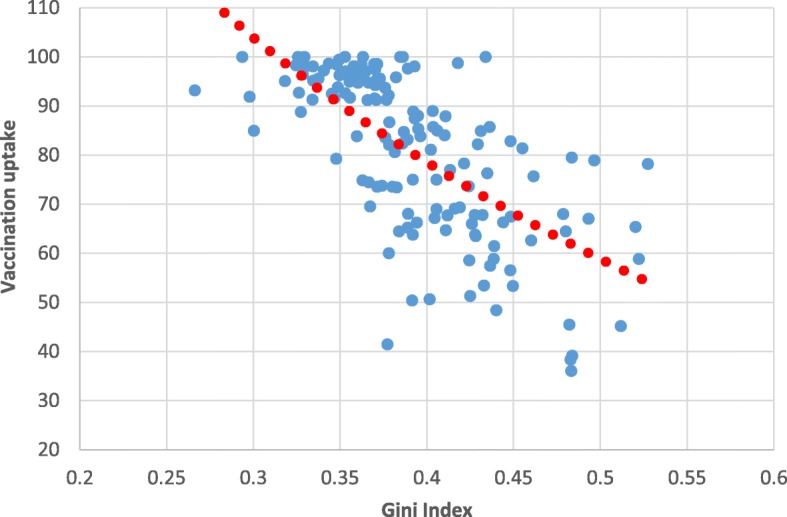
OPV Vaccination Uptake, by Gini Index (Municipality). Source: Ministry of Health, processed by the authors

Figure 2 shows the correlation between vaccination uptake and the income inequality index of the municipality where the mother-and-child clinic is situated. A negative correlation was found between these variables, meaning that the greater the income inequality in the municipality where the mother-and-child clinic is situated, the lower the vaccination uptake is. In other words, insofar as the mother-and-child clinic is situated in a municipality typified by greater income inequality, vaccination uptake in that municipality is lower.

**Fig. 3:**
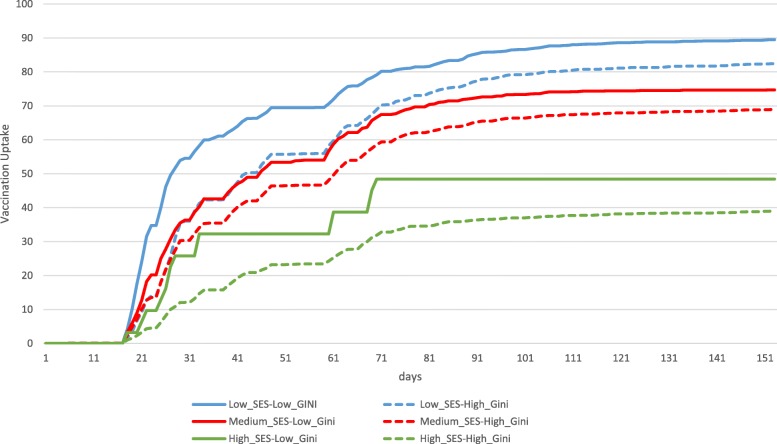
Longitudinal OPV Vaccination Uptake a Gini Index Comparison as a Function of SES, August–December 2013 (Municipality). Source: Ministry of Health, processed by the authors

### Data analysis

The average vaccination uptake among municipalities in low-SES and low income-inequality levels was nearly 90% at the end of the campaign period. Among municipalities in low-SES areas typified by high income inequality indices, vaccination uptake was nearly 80%. Average vaccination uptake at municipalities in medium-SES areas and low income-inequality indices was around 75% at the end of the campaign period. Among municipalities in medium-SES areas that were typified by high income inequality, vaccination uptake was approximately 70%. Average vaccination uptake at municipalities in high-SES areas, at the end of the campaign period, was 50% in municipalities with low income inequality and roughly 40% in municipalities with high income inequality (see Fig. 3).

## Discussion

Compliance with or opposition to vaccination falls within the limits of state power in the private sphere (like family, religion, and health beliefs) that is often emphasized by ethnic tensions [24]. Thus, Israel’s 2013 polio vaccination campaign is intricately linked to, and must be understood in the context of communities’ social standing [24]. Understanding the decision to vaccinate in larger social contexts, in contrast to viewing vaccination as an individualized decision alone, is not unique to Israel [24]. Policymakers are encouraged to strengthen principles of solidarity in their efforts to eradicate SES and equality disparities in healthcare (https://www.cambridge.org/core/books/solidarity-in-biomedicine-and-beyond/067DC974D204F6EDE679816213433456). The overall success of the campaign may be partly attributed to the Israeli focus on safety of the family and solidarity for others as motives for vaccination [24]. Nevertheless, the variance in vaccine uptake rates shows that Israel has differing vaccination uptake among different SES clusters of society.

The question of vaccination uptake patterns as a function of the economic inequality index has not been examined thus far; this is the first specific contribution of this study. Vaccination uptake was significantly higher in municipalities with lower income inequality indices than in municipalities where the index was high.

The study also shows the ability to examine how local (municipal) vaccine uptake is affected, both separately and jointly, by the average SES level and GINI inequality index of a community. Vaccination uptake varied as a function of the connection between a mother-and-child clinic and a given SES and income-inequality index. The vaccination uptake rate was found to be a negative function of the SES level, i.e., as the SES level rose, the uptake rate fell. The results of this study suggests that SES is not the only inequality index that relates to vaccination uptake. The Gini inequality index was found to be a consistent indicator of vaccination uptake when SES is controlled for. Polio vaccination uptake was consistently higher among populations with a low Gini inequality index in comparison to populations with medium and high Gini inequality indices, regardless of SES status.

The integration of these two leading socioeconomic indices shows that the vaccination uptake rate was highest among populations of low SES and low Gini inequality index, followed respectively by low SES/high Gini inequality index, medium SES/low Gini inequality index, medium SES/high Gini inequality index, high SES/low Gini inequality index, and high SES/high Gini inequality index. This outcome is particularly surprising in view of its inversion of the explanatory tendency. Thus, insofar as the socioeconomic index, manifested in SES, reflects a higher and stronger social level in the geographic environs of the medical service, the vaccination rate in the same area falls. In contrast, as the inequality index in the area falls, the vaccination uptake rate in the vicinity rises.

How health inequalities influence economic, social, cultural and political aspects of public heatlh continues to plague researchers, policymakers, and decision makers [25, 26]. How solidarity contributes to such influences in the context of vaccination uptake is an applied example [20, 24]. Prainsack and Buyx define solidarity as the “willingness to carry costs to assist others with whom a person recognizes sameness or similarity in at least one relevant aspect”. In the field of public health, solidarity is a value that is consistently used to justify stronger involvement of state authority involvement in reducing health inequities [27]. Appeals to solidarity raise questions about where boundaries between individual, family, community, and society responsibility in reducing health inequalities should be drawn [27]. How health inequalities are measured also play a role in determining the place of solidarity in public health.

Solidarity helps to close health disparities. Prainsack and Buyx define solidarity in “its most bare-boned form” as “shared practices reflecting a collective commitment to carry ‘costs’ (financial, social, emotional, and otherwise) to assist others” [27, 28]. The act of solidarity, according to Prainsack and Buyx, is “embodied and enacted rather than merely ‘felt’.” Vaccination and solidarity are tightly intertwined concepts in the field of public health. Because it cannot be assumed that people will accept the potential risk of vaccine side-effects due to the abstract thought that a pandemic might place them in an at-risk group at some point in their lives, vaccination campaigns generally originate in state authorities [27]. While individuals weigh personal risks and benefits of vaccines, governments think of vaccinations in terms of “herd immunity” and consider individuals’ gain as an added benefit of vaccinating for the greater public good [24].

During the 2013 polio vaccination campaign in Israel, the need for collective action that would not directly benefit the individual became very apparent [24]. Although the ethic of solidarity and a renewed sense of investment in others’ welfare cannot be legislated into existence, it can be cultivated by human endeavor, specifically in the form of education [29]. Concepts of “society,” “solidarity,” and “individualism” are used in different ways by different actors to persuade and evoke compliance, simultaneously affecting public and policymaker understanding [24].

Collecting data on vaccination uptake at a highly specific level of resolution and not only at the district level, in a manner that includes SES, aids healthcare system policymakers in establishment-targeted intervention programs to increase vaccination uptake. This study suggests a correlation between solidarity and the Gini inequality index and stresses not only the need for future research to contextualize its findings, but for policy makers to account for SES and solidarity in implementing vaccination policy. Incorporating SES and solidarity in vaccination policy was a practice used during 2018 measles outbreaks in Israel [30]. This study stresses the need to understanding the value of the Gini inequality index when incorporating solidarity and SES into decisions regarding vaccination policy.

## Conclusions

This study shows that in communities that are stronger socio-economically, there is less inclination for parents to ensure that their children are vaccinated. Additionally, in municipalities where there are smaller economic gaps and community members are more similar in SES status, there is a greater tendency to vaccinate their children against polio.

This finding also touches on issues “free-riding”, suggesting that free riders may be found more frequently in communities with larger gaps of inequality (higher GINI indices), since this study’s suggests that in communities with lower inequalities, vaccination uptake is higher. Due to this study, policymakers will be able to reevaluate their resource allocation regarding vaccination campaigns among different communities based on the suggested correlation suggested between the Gini coefficient and SES status by this paper.

Vaccination rates were significantly higher among the Bedouin population than in any district countrywide. This finding provides further support for the “paradox” in regard to vaccination uptake and health inequalities: despite higher health inequalities present in areas of low SES, vaccination rates were higher than populations with lower health inequalities and high SES. Nevertheless, it is important to note that there are implications of potential income disparities that may describe propensity to vaccinate infants. This contributes to a limitation of this study—the potential to overlook relevant nuances contributing to vaccination uptake of children. Vaccination and children are sensitive subjects on their own when it comes to health behavior. Anti-vaccination campaigns often gain heightened traction when appealing to false claims stating that vaccines cause disproportionate harm to children [31]. The case of vaccinating children during the 2013 polio outbreak in Israel in addition to the “urgency” that results from containing disease outbreaks adds further complexity to this analysis of vaccination uptake. With this in mind, the correlation between solidarity and the Gini inequality index has been implied in other academic spheres such as immigration and welfare studies [32]. However, the correlation has yet to be explicitly suggested within public health and health policy.

Public health professionals promoting vaccine programs need to make specially-designed efforts both in localities with high average income and in localities with a high level of income diversity/inequality. Such practice will better utilize funds, resources, and manpower dedicated to increasing vaccination uptake across varying populations and communities.

This study speaks to a specific time, place, and case study. The 2013 polio outbreak in Israel suggests a unique relationship between socioeconomic and equality indices such as SES and the Gini inequality coefficient to notions of solidarity. It builds upon previous research conducted in Israel suggesting a relationship between vaccination uptake and SES status [20]. The question of role of nationality and locality size in explaining differences among localities in vaccination uptake therefore arises. In exploring this notion, economists, municipalities, statisticians, and public health professionals would meaningfully contribute to applying the evaluation of SES and the Gini coefficient in creating revised vaccination campaigns.

Future research on the correlation between the solidarity and the Gini inequality index (for example, in terms of health behavior) is crucial in order to better contextualize and apply this study’s findings in order to better improve vaccination campaigns as well. The correlation between the GINI coefficient and solidarity has been explored in other fields besides public health, including migration, welfare, and economics [32, 33]. The results of this study promote further investigations into the correlation between solidarity and different aspects of society and culture. In the meantime, stakeholders and decision-makers are urged to incorporate SES and solidarity into vaccination policy and health policy in general.

## Data Availability

Unfortunately, the data cannot be shared because, at the present writing, the Ministry of Health database is not open to the public.

## Footnotes

1 89.2% in the north, 70.8% in Haifa, 78.3% in Jerusalem, 68.9% in the Center, and 83.2% in the South.

## Abbreviations

IPV: Inactivated poliovirus vaccine
OPV: Oral poliovirus *vaccine*
*SES*: Socioeconomic status
WPV1: Wild polio virus 1
